# Supplementary material for: Mechanical properties measured by atomic force microscopy define health biomarkers in ageing C. elegans
Source: Nat Commun. 2020 Feb 25;11:1043. doi: 10.1038/s41467-020-14785-0 (PMC7042263; doi:10.1038/s41467-020-14785-0)
Supplement: Supplementary file 1 — Supplementary Information [file 41467_2020_14785_MOESM1_ESM.pdf]

Mechanical properties measured by Atomic Force Microscopy define health biomarkers in ageing *C. elegans*

Essmann et al.

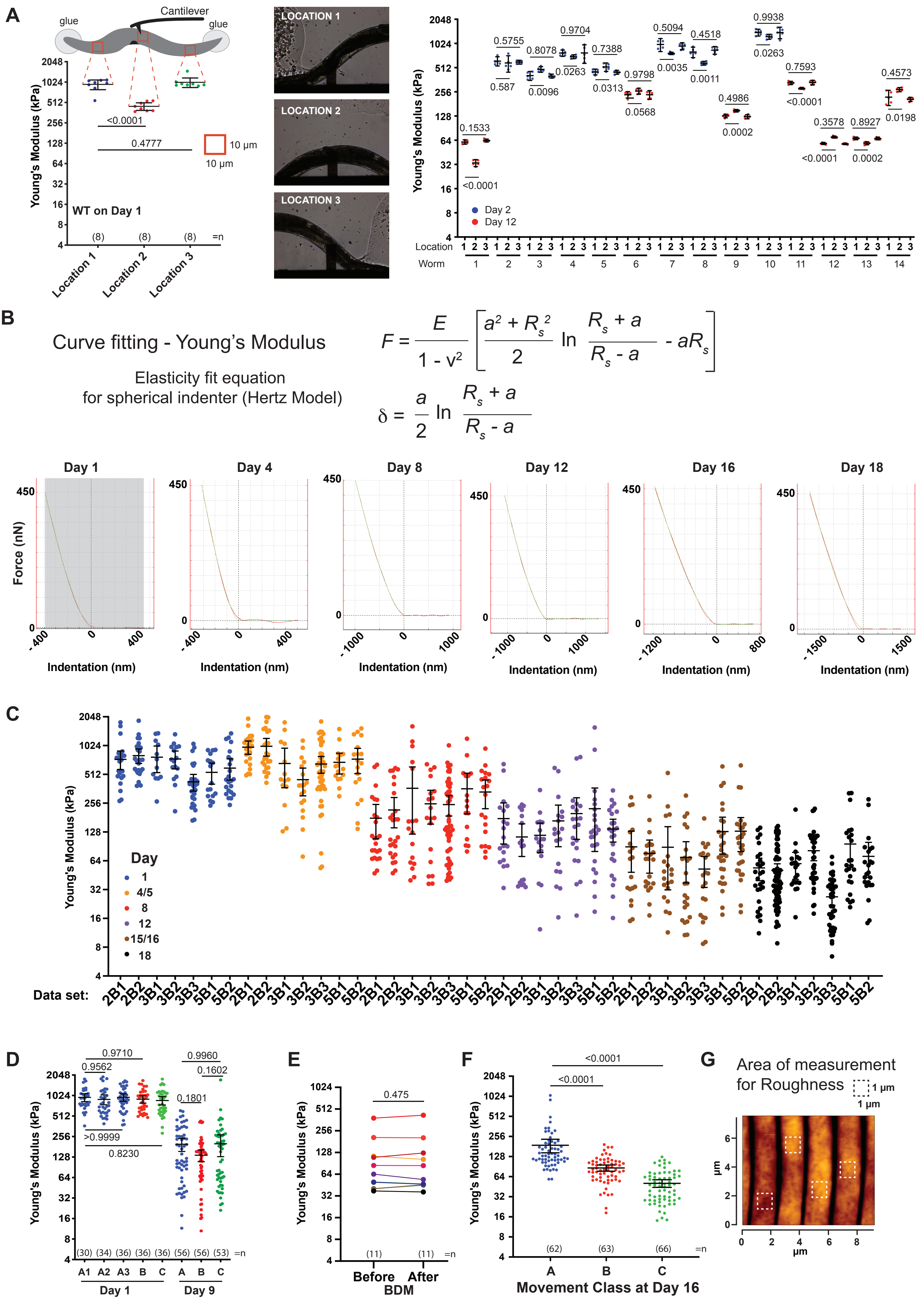
A
Cantilever
glue
glue
WT on Day 1
Young's Modulus (kPa)
<0.0001
0.4777
10 µm
10 µm
Location 1
Location 2
Location 3
(8) (8) (8) =n
LOCATION 1
LOCATION 2
LOCATION 3
Day 2
Day 12
Location
Worm
B
Curve fitting - Young's Modulus
Elasticity fit equation
for spherical indenter (Hertz Model)
$F = \frac{E}{1 - \nu^2}\left[\frac{a^2 + R_s^2}{2} \ln \frac{R_s + a}{R_s - a} - aR_s\right]$
$\delta = \frac{a}{2} \ln \frac{R_s + a}{R_s - a}$
Day 1
Day 4
Day 8
Day 12
Day 16
Day 18
Force (nN)
Indentation (nm)
C
Young's Modulus (kPa)
Day
1
4/5
8
12
15/16
18
Data set:
D
Young's Modulus (kPa)
0.9710
0.9562
>0.9999
0.8230
0.9960
0.1602
0.1801
(30) (34) (36) (36) (36) (56) (56) (53) =n
A1 A2 A3 B C A B C
Day 1
Day 9
E
Young's Modulus (kPa)
0.475
(11) (11) =n
Before After
BDM
F
Young's Modulus (kPa)
<0.0001
<0.0001
(62) (63) (66) =n
A B C
Movement Class at Day 16
G
Area of measurement
for Roughness
1 µm
1 µm
µm
µm

Supplementary Figure 1. Measuring mechanical properties of *C. elegans*.

A) Mechanical properties as Young's Modulus (YM, kPa) from 3 different locations of wild type *C. elegans* (location 1 (neck), location 2 (mid-body), location 3 (hip)). Left panel- Example measurement and worm scheme; Mid-panel- Example of brightfield images; Right-panel- additional YM's measurements for independent worms in 3 distinct locations at day 2 and 12. 5-8 measurements were taken at each location within a 10 x10 μm field of view. Two-tailed unpaired t-test for statistical comparison of location 1 to location 2 or 3 for each worm. **B)** Curve fitting of individual force-indentation curves to the calculation of YM values. Hertz fit equation (upper panel) and examples of the overlay of the Hertz fit (green curve) to an experimental force curve (red curve) of a WT worm at the indicated age. The grey area in day 1 panel indicates the curve fit range. **C)** Display of biological variability within AFM measurements obtained from several datasets contained in Figure 2B, 3B and 5B. Mechanical properties as YM (kPa) of WT *C. elegans* control at day 1, 4/5, 8, 12, 15/16 and 18 as independent experimental trials. Error bars indicate 95% CI. **D)** Variability within technical replicates for AFM measurements. Mechanical properties as YM (kPa) from 1-day or 9-days old WT *C. elegans* from different plates grown in parallel under same media and temperature conditions seeded with the culture of the same bacterial colony (A1-3) or seeded with a culture obtained from growing different individual bacterial colonies (A, B, C) from an isogenic OP50 population streaked on an LB plate. Error bars indicate 95% CI. One-way ANOVA Tukey's multiple comparison test for statistical comparison between the replicates of day 1 or day 9. **E)** Mechanical properties as YM (kPa) of 11 old worms before (left) and after (right) 1h-treatment with paralyzing agent BDM. Two-tailed paired t-test for statistical comparison of before and after BDM treatment. **F)** Mechanical properties as YM (kPa) from wild type *C. elegans* at age of 16 days grouped according to their movement class phenotype when prodded: fully moving (A, blue), moving head or tail (B, red) or not moving (C, green). Error bars indicate 95% CI. Two-tailed unpaired t-test for statistical comparison of A to B or C. **G)** Roughness measurements of the cuticle. White dotted squares indicate 4 independent areas of measurements within the annuli region of a topographical image.

n, represented above the graph, show number of biologically independent worm samples and for a summary of YM values and additional statistics for independent trials see Supplementary Data 1. Source data are provided as a Source Data file.

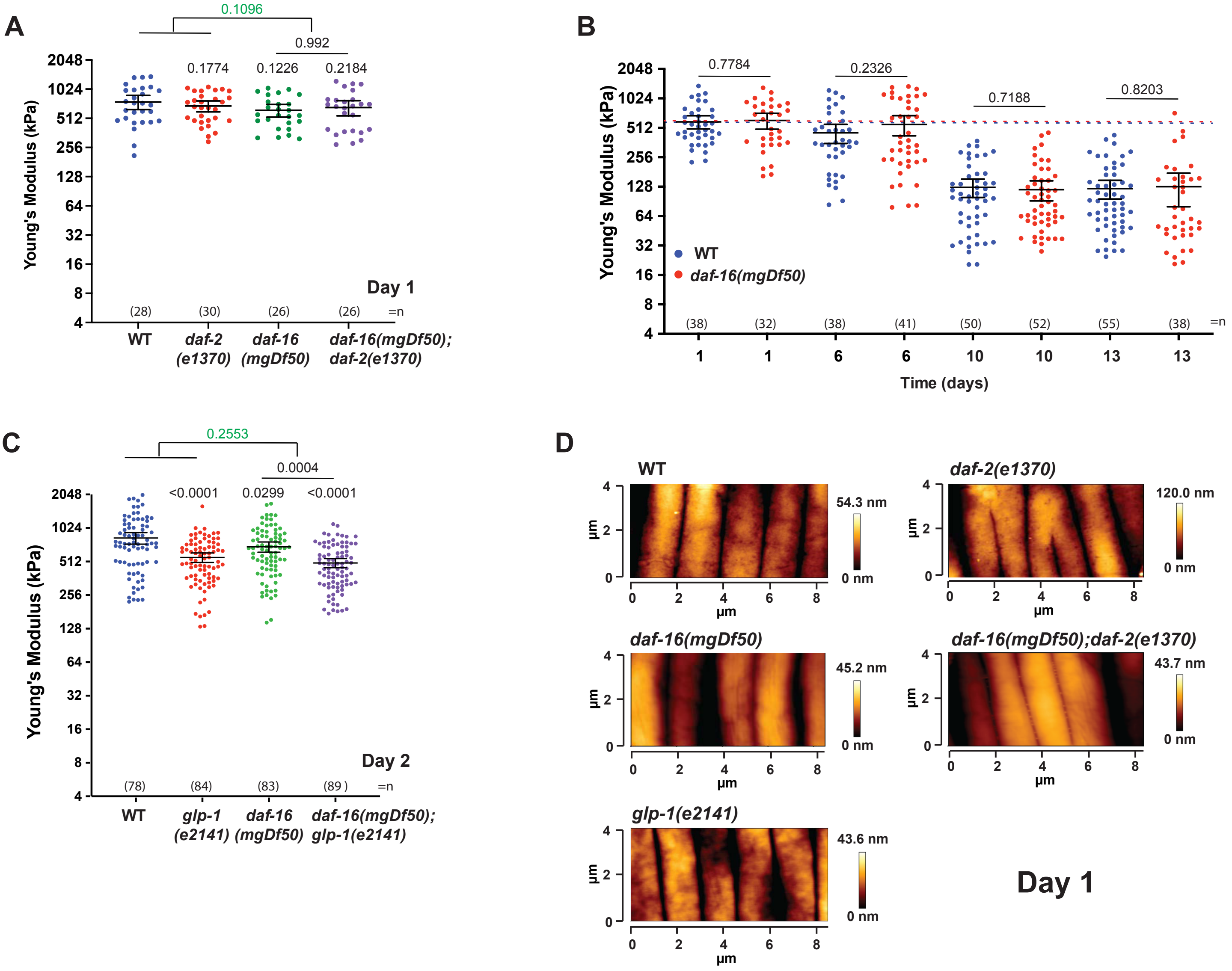
A
0.1096
0.992
0.1774 0.1226 0.2184
Young's Modulus (kPa)
2048
1024
512
256
128
64
32
16
8
4
Day 1
(28) (30) (26) (26) =n
WT
daf-2 (e1370)
daf-16 (mgDf50)
daf-16(mgDf50); daf-2(e1370)
B
0.7784 0.2326 0.7188 0.8203
WT
daf-16(mgDf50)
(38) (32) (38) (41) (50) (52) (55) (38) =n
1 1 6 6 10 10 13 13
Time (days)
C
0.2553
0.0004
<0.0001 0.0299 <0.0001
Day 2
(78) (84) (83) (89) =n
WT
glp-1 (e2141)
daf-16 (mgDf50)
daf-16(mgDf50); glp-1(e2141)
D
WT
54.3 nm
0 nm
daf-2(e1370)
120.0 nm
0 nm
daf-16(mgDf50)
45.2 nm
0 nm
daf-16(mgDf50);daf-2(e1370)
43.7 nm
0 nm
glp-1(e2141)
43.6 nm
0 nm
µm
Day 1

Supplementary Figure 2. Stiffness and cuticle topography of CGCM strain, insulin and *glp-1* mutants.

A) Mechanical properties as YM (kPa) of wild type WT (blue), *daf-2* (red), *daf-16* (green) and *daf-16;daf-2* (purple) mutant *C. elegans* at chronological age of day 1. Error bars indicate 95% CI. Two-way ANOVA Tukey's multiple comparison test for statistical comparison and interaction of terms (green). **B)** Longitudinal study of mechanical properties as YM (kPa) comparing wild-type WT (blue) and *daf-16* mutant (red) *C. elegans* until mean lifespan of *daf-16* (D13). Error bars indicate 95% CI, dotted lines mark mean YM at day 1 for WT and *daf-16*. Two-tailed unpaired t-test for statistical comparison of WT to *daf-16*. **C)** Mechanical properties as YM (kPa) of wild type WT (blue), *glp-1* (red), *daf-16* (green) and *daf-16;glp-1* (purple) mutant *C. elegans* at chronological age of day 2. Error bars indicate 95% CI. Two-way ANOVA Tukey's multiple comparison test for statistical comparison and interaction of terms (green). **D)** Representative AFM cuticle topography images of young adult day 1 WT, *daf*-2, *daf*-16, *day-16;daf-2*, *and glp-1* mutant *C. elegans.*

n, represented above the graph show number of biologically independent worm samples and for a summary of YM values and additional statistics for independent trials see Supplementary Data 1. Source data are provided as a Source Data file.

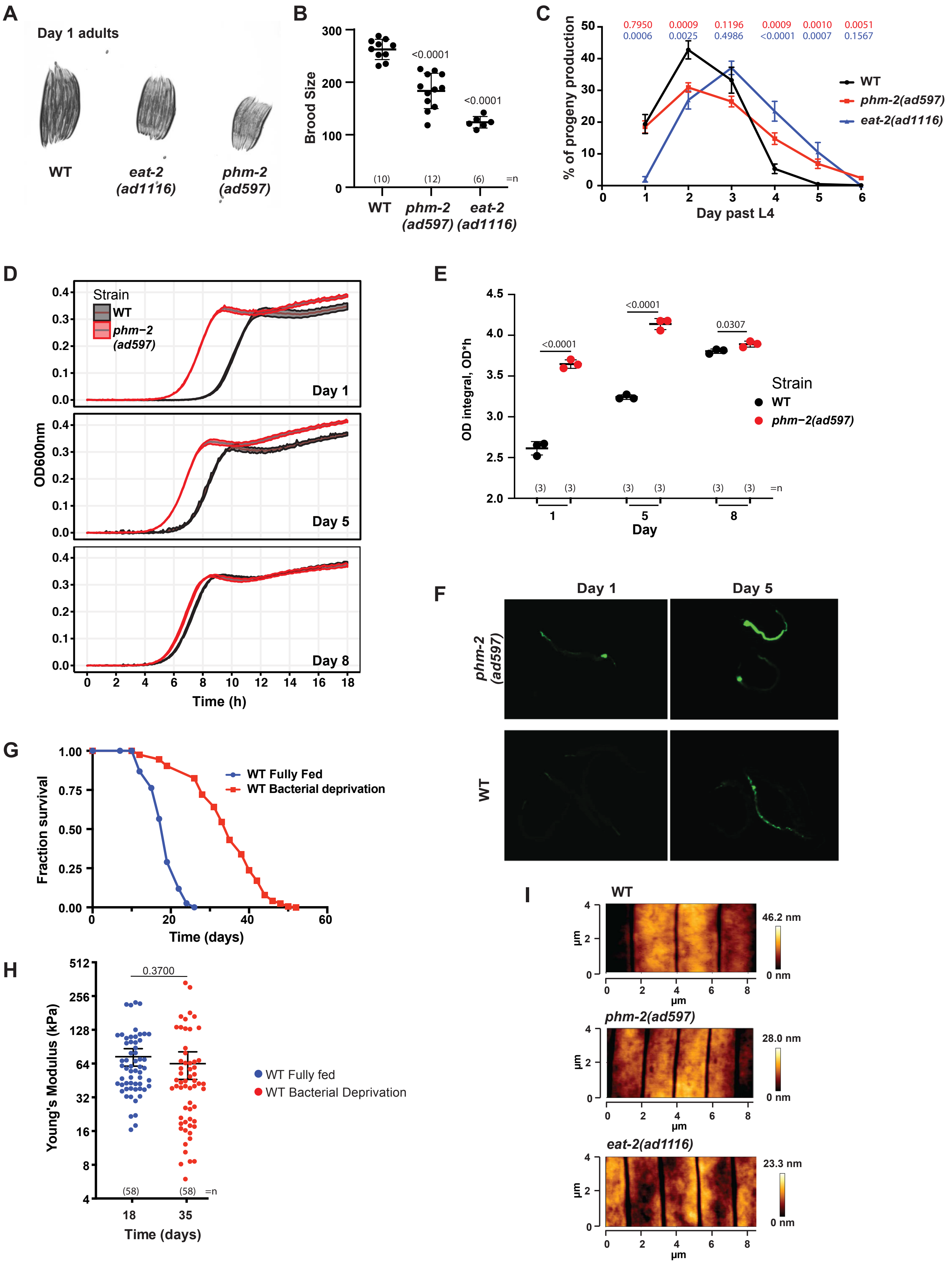
A
Day 1 adults
WT
eat-2 (ad1116)
phm-2 (ad597)
B
Brood Size
300
200
100
0
<0.0001
<0.0001
(10)
(12)
(6)
=n
WT
phm-2 (ad597)
eat-2 (ad1116)
C
% of progeny production
0.7950 0.0009 0.1196 0.0009 0.0010 0.0051
0.0006 0.0025 0.4986 <0.0001 0.0007 0.1567
WT
phm-2(ad597)
eat-2(ad1116)
Day past L4
D
Strain
WT
phm−2 (ad597)
OD600nm
Day 1
Day 5
Day 8
Time (h)
E
OD integral, OD*h
<0.0001
<0.0001
0.0307
Strain
WT
phm−2(ad597)
(3) (3) (3) (3) (3) (3) =n
Day
F
Day 1
Day 5
phm-2 (ad597)
WT
G
Fraction survival
WT Fully Fed
WT Bacterial deprivation
Time (days)
H
Young's Modulus (kPa)
0.3700
(58)
(58)
=n
WT Fully fed
WT Bacterial Deprivation
Time (days)
I
WT
46.2 nm
0 nm
phm-2(ad597)
28.0 nm
0 nm
eat-2(ad1116)
23.3 nm
0 nm
μm

Supplementary Figure 3. Physiological characterisation of dietary restriction mutants and conditions.

A) Bright-field image showing size and appearance of young day 1 adult WT (left), *eat-2* (middle) or *phm-2* (right) mutant *C. elegans.* **B)** Histogram shows brood size of wild-type WT, *phm-2* or *eat-2* mutant. Two-tailed unpaired t-test for statistical comparison of WT to mutants. **C)** Daily fecundity as a proportion of total fecundity for each *C. elegans* genotype. Multiple t-tests corrected for multiple comparisons using the Benjamini-Hochberg adjustment comparing WT to *phm-2* (red) or WT to *eat-2* (blue). **D)** Bacterial growth curves over an 18-hour period obtained from bacterial colonisation tests for WT and *phm-2* mutant *C. elegans* at different ages. Each curve is the sum of three independent biological replicates. Shaded areas represent S.D. **E)** Quantification of the area under the curve for each genotype per age measured from **D**. Error bars represent S.D. Two-tailed unpaired t-test for statistical comparison of WT to mutant at the indicated day. **F)** Representative images of gut bacterial colonisation of WT and *phm-2* mutant *C. elegans* with OP50 expressing GFP. **G)** Lifespan curves of wild type WT fully-fed (blue) and WT *C. elegans* under bacterial deprivation (BD) (red) (n = 98 and 165 respectively; log rank test $p < 0.001$ vs fully fed WT), and **H)** mechanical properties as Young's Modulus (kPa) at mean lifespan (D18 fully fed, D35 bacterial deprivation). Error bars indicate 95% CI. Two-tailed unpaired t-test for statistical comparison. **I)** Representative AFM cuticle topography images of day 1 young adult *eat-2* or *phm-*2 mutant *C. elegans*

n represented above the graph, show number of biologically independent worm samples; For lifespan measurements, *n* represents the number of worms scored as dead. For a summary of YM values and additional statistics for independent trials see Supplementary Data 1 and for a summary of worm lifespan trials and statistical comparison between genotypes see Supplementary Data 2. Source data are provided as a Source Data file.

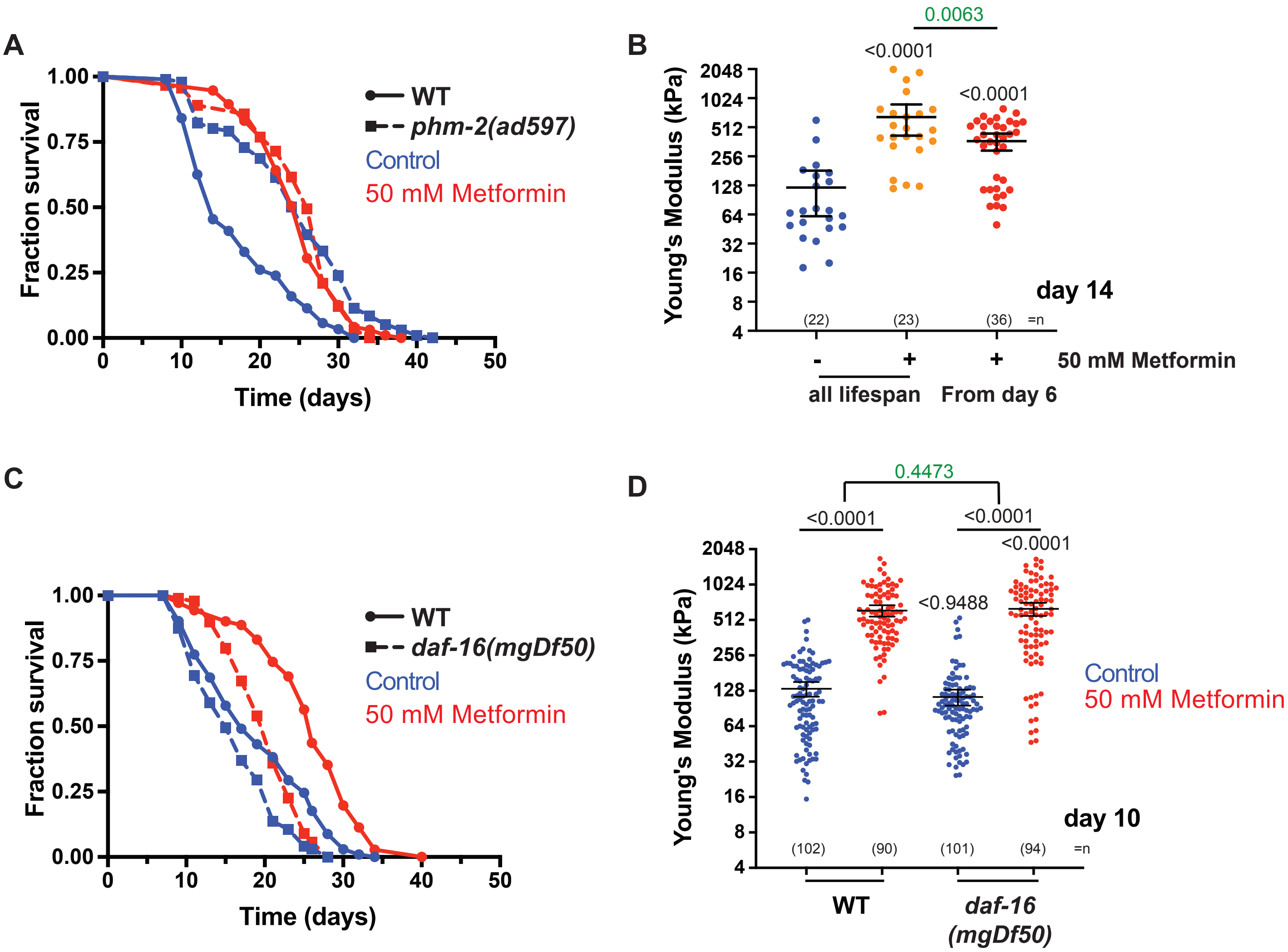
A
Fraction survival
1.00
0.75
0.50
0.25
0.00
0 10 20 30 40 50
Time (days)
WT
phm-2(ad597)
Control
50 mM Metformin
B
0.0063
<0.0001
<0.0001
Young's Modulus (kPa)
2048
1024
512
256
128
64
32
16
8
4
day 14
(22) (23) (36) =n
- + + 50 mM Metformin
all lifespan From day 6
C
Fraction survival
Time (days)
WT
daf-16(mgDf50)
Control
50 mM Metformin
D
0.4473
<0.0001
<0.0001
<0.0001
<0.9488
Young's Modulus (kPa)
Control
50 mM Metformin
day 10
(102) (90) (101) (94) =n
WT
daf-16
(mgDf50)

Supplementary Figure 4. Metformin and host genetics interactions regulating lifespan and stiffness.

A) Lifespan curve of WT (full line) and *phm-2* mutant (dotted line) *C. elegans* treated with 0 mM metformin (blue) or 50 mM metformin (red) (*n* = 88, 95, 96 and 91, respectively; log rank test $p < 0.001$ vs WT 0 mM). **B)** Mechanical properties as Young's Modulus (YM; kPa) at chronological age day 14 of wild type *C. elegans* untreated (blue), treated with 50 mM metformin (yellow) and treated with 50 mM metformin from day 6 of adulthood (red). Error bars indicate 95% CI. Two-tailed unpaired t-test for statistical comparison of untreated with treated (black), or between treated conditions (green). **C)** Lifespan curve of WT (full line) and *daf-16* mutant (dotted line) *C. elegans* treated with 0 mM metformin (blue) or 50 mM metformin (red) (*n* = 102, 71, 95 and 89, respectively; log rank test $p < 0.001$, $p < 0.001$ and $p = 0.4398$ vs WT 0 mM). D) Mechanical properties as YM (kPa) of WT and *daf-16* mutant *C. elegans* untreated (blue) or metformin-treated (red) at chronological age of day 11. Error bars indicate 95% CI. Two-way ANOVA Tukey's multiple comparison test for statistical comparison and interaction of terms (green).

n represented above the graph, show number of biologically independent worm samples; For lifespan measurements, *n* represents the number of worms scored as dead. For a summary of YM values and additional statistics for independent trials see Supplementary Data 1 and for a summary of worm lifespan trials and statistical comparison between genotypes see Supplementary Data 2. Source data are provided as a Source Data file.

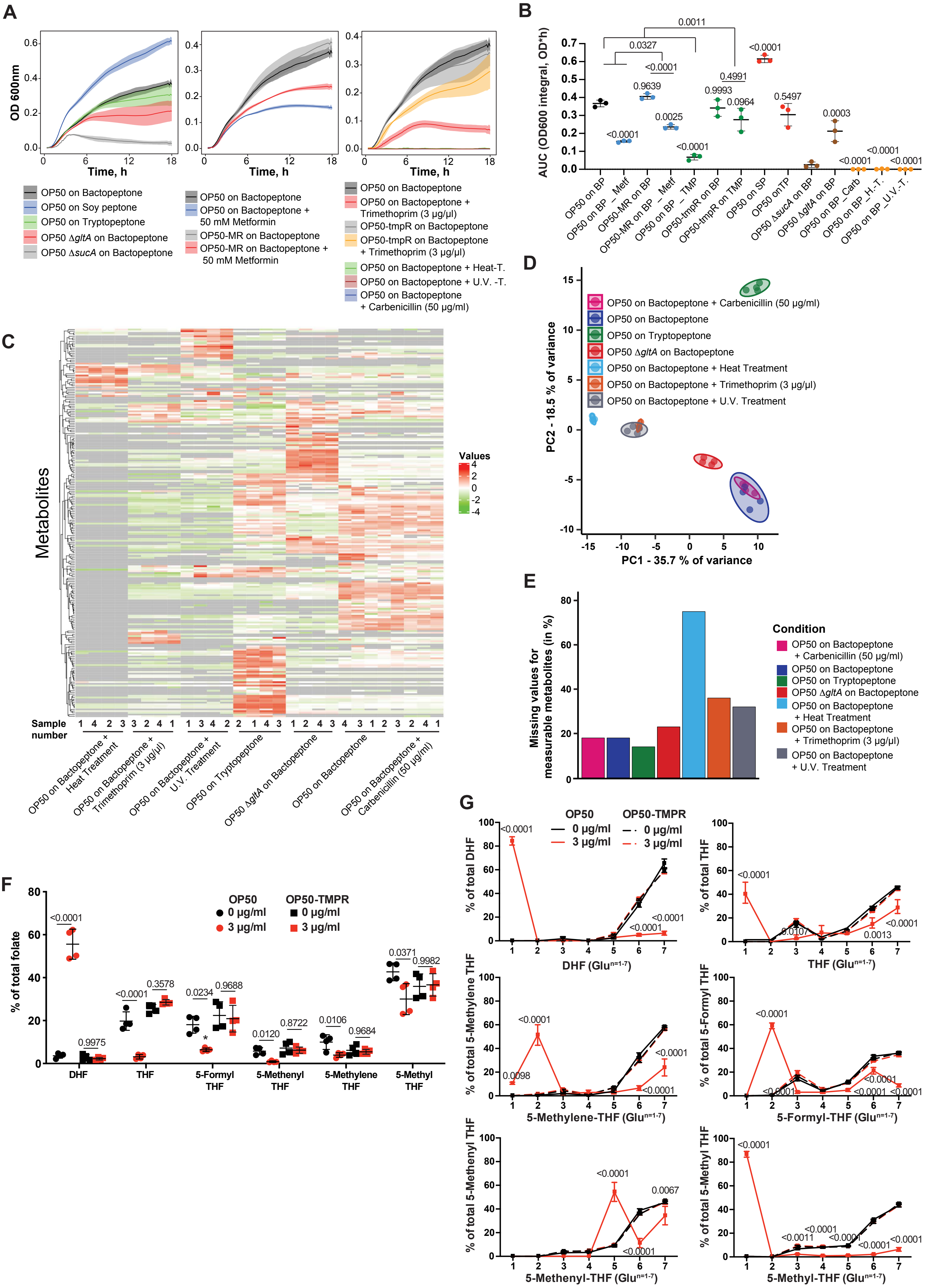
A
OD 600nm
Time, h
OP50 on Bactopeptone
OP50 on Soy peptone
OP50 on Tryptopeptone
OP50 ΔgltA on Bactopeptone
OP50 ΔsucA on Bactopeptone
OP50 on Bactopeptone
OP50 on Bactopeptone + 50 mM Metformin
OP50-MR on Bactopeptone
OP50-MR on Bactopeptone + 50 mM Metformin
OP50 on Bactopeptone
OP50 on Bactopeptone + Trimethoprim (3 µg/µl)
OP50-tmpR on Bactopeptone
OP50-tmpR on Bactopeptone + Trimethoprim (3 µg/µl)
OP50 on Bactopeptone + Heat-T.
OP50 on Bactopeptone + U.V. -T.
OP50 on Bactopeptone + Carbenicillin (50 µg/ml)
B
AUC (OD600 integral, OD*h)
0.0011
0.0327
<0.0001
0.9639
<0.0001
0.0025
<0.0001
0.9993
0.4991
0.0964
<0.0001
0.5497
0.0003
<0.0001
<0.0001
<0.0001
OP50 on BP
OP50 on BP _ Metf
OP50-MR on BP
OP50-MR on BP _ Metf
OP50 on BP _ TMP
OP50-tmpR on BP
OP50-tmpR on TMP
OP50 on SP
OP50 onTP
OP50 ΔsucA on BP
OP50 ΔgltA on BP
OP50 on BP _Carb
OP50 on BP _H.-T.
OP50 on BP _U.V.-T.
C
Metabolites
Values
Sample number
OP50 on Bactopeptone + Heat Treatment
OP50 on Bactopeptone + Trimethoprim (3 µg/µl)
OP50 on Bactopeptone + U.V. Treatment
OP50 on Tryptopeptone
OP50 ΔgltA on Bactopeptone
OP50 on Bactopeptone
OP50 on Bactopeptone + Carbenicillin (50 µg/ml)
D
OP50 on Bactopeptone + Carbenicillin (50 µg/ml)
OP50 on Bactopeptone
OP50 on Tryptopeptone
OP50 ΔgltA on Bactopeptone
OP50 on Bactopeptone + Heat Treatment
OP50 on Bactopeptone + Trimethoprim (3 µg/µl)
OP50 on Bactopeptone + U.V. Treatment
PC2 - 18.5 % of variance
PC1 - 35.7 % of variance
E
Missing values for measurable metabolites (in %)
Condition
OP50 on Bactopeptone + Carbenicillin (50 µg/ml)
OP50 on Bactopeptone
OP50 on Tryptopeptone
OP50 ΔgltA on Bactopeptone
OP50 on Bactopeptone + Heat Treatment
OP50 on Bactopeptone + Trimethoprim (3 µg/µl)
OP50 on Bactopeptone + U.V. Treatment
F
% of total folate
OP50
0 µg/ml
3 µg/ml
OP50-TMPR
0 µg/ml
3 µg/ml
<0.0001
0.9975
<0.0001
0.3578
0.0234
0.9688
0.0120
0.8722
0.0106
0.9684
0.0371
0.9982
DHF
THF
5-Formyl THF
5-Methenyl THF
5-Methylene THF
5-Methyl THF
G
OP50
0 µg/ml
3 µg/ml
OP50-TMPR
0 µg/ml
3 µg/ml
% of total DHF
DHF (Glu$^{n=1-7}$)
% of total THF
THF (Glu$^{n=1-7}$)
% of total 5-Methylene THF
5-Methylene-THF (Glu$^{n=1-7}$)
% of total 5-Formyl THF
5-Formyl-THF (Glu$^{n=1-7}$)
% of total 5-Methenyl THF
5-Methenyl-THF (Glu$^{n=1-7}$)
% of total 5-Methyl THF
5-Methyl-THF (Glu$^{n=1-7}$)

Supplementary Figure 5. Antibiotic, nutritional and genetic modulation of bacterial growth, respiration and metabolism.

A) Bacterial growth curves for *E. coli* in diverse growth conditions and treatments. Each graph represents the average of 3 independent biological replicates. Shaded areas represent S.D. **B)** Area under the curve (AUC) measurements of bacterial growth in diverse growth conditions and treatments. Each condition represents the average of 3 independent biological replicates. Error bars represent S.D. Two-way ANOVA test with false discovery rate method (FDR) were used for statistical comparison of treatment/condition to OP50 on Bactopeptone. **C)** Heat-map of untargeted metabolomics data of OP50 under different growth conditions and/or treatments displaying replicability between 4 independent biological replicates. Red (higher) to green (lower) gradient represents abundance of a specific metabolite in a specific condition. Grey represent missing values N.D. (see material and methods and Supplementary Data 3) **D)** PCA plot of untargeted metabolomics data showing the effect of distinct interventions on the metabolome of *E. coli* OP50. Missing N.D. values were substituted by a value of 2E-52. **E)** Bar graph plot displaying the percentage of metabolites with N.D. values over a total of 228 metabolites with potential detection in diverse conditions. **F)** Trimethoprim treatment (3 µg/ml) impairs folate cycle homeostasis in OP50 but not OP50-TMPR over-expressing a dihydrofolate reductase cassete. DHF = dihydrofolate; THF = tetrahydrofolate. The value for each metabolite is the ratio between the sum of the values for the different glutamate side chains (1-7) and the sum of all metabolites measured. Each value is the result of the measurement of 4 independent biological replicates. Error bars represent S.D. Two-way ANOVA Tukey's multiple comparison test for statistical comparison of untreated vs treated of each sample condition. **G)** Trimethoprim treatment (3 µg/ml) impairs polyglutamylation of folate metabolites in OP50 but not OP50-TMPR. Polyglutamylation profiles for each metabolite are shown. Error bars represent S.E.M. Two-way ANOVA Tukey's multiple comparison tests were used for statistical comparison. Source data are provided as a Source Data file.

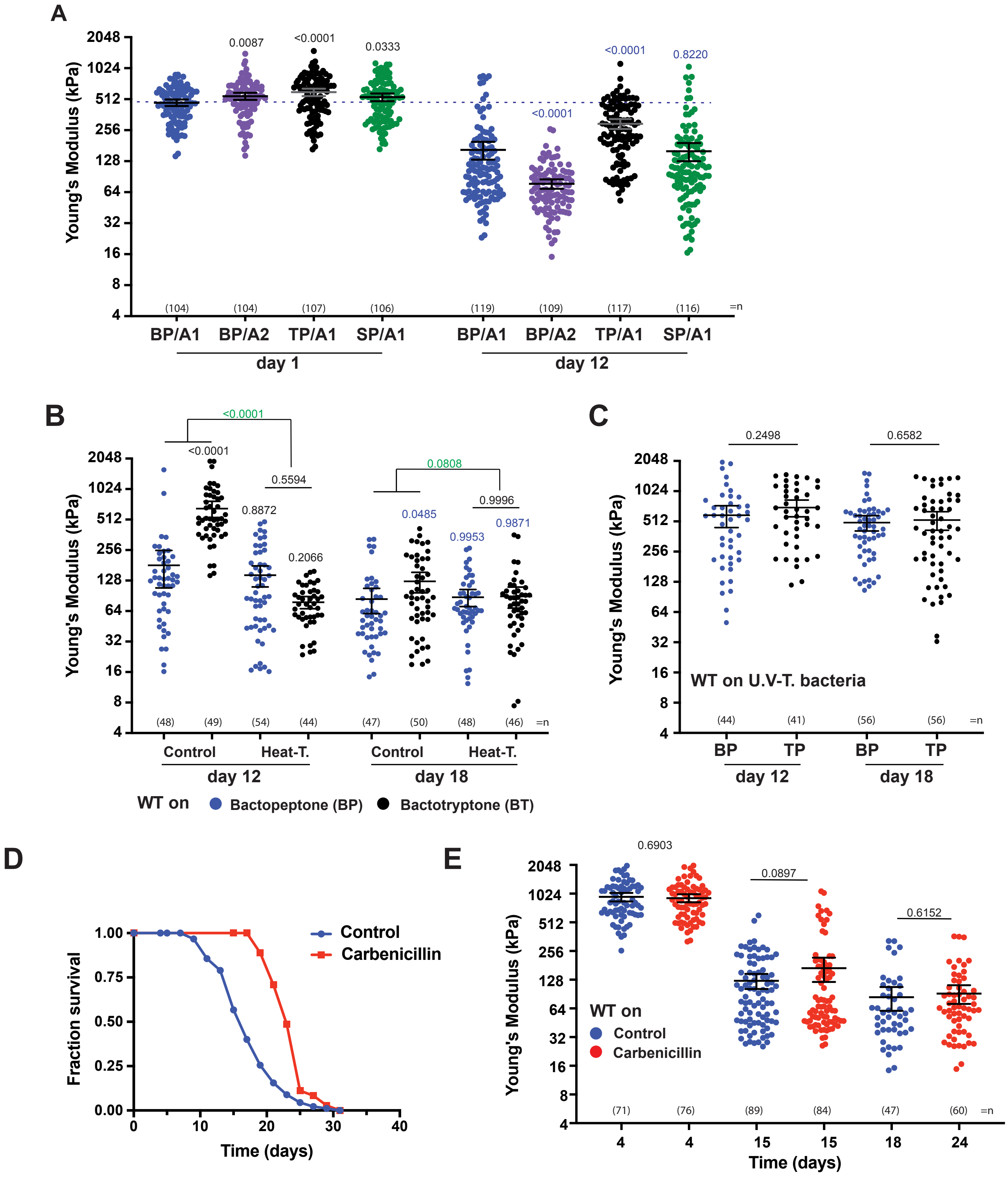
A
Young's Modulus (kPa)
2048
1024
512
256
128
64
32
16
8
4
0.0087
<0.0001
0.0333
<0.0001
0.8220
<0.0001
(104)
(104)
(107)
(106)
(119)
(109)
(117)
(116)
=n
BP/A1
BP/A2
TP/A1
SP/A1
BP/A1
BP/A2
TP/A1
SP/A1
day 1
day 12
B
<0.0001
<0.0001
0.5594
0.8872
0.2066
0.0808
0.9996
0.0485
0.9953
0.9871
(48)
(49)
(54)
(44)
(47)
(50)
(48)
(46)
=n
Control
Heat-T.
Control
Heat-T.
day 12
day 18
WT on
Bactopeptone (BP)
Bactotryptone (BT)
C
0.2498
0.6582
WT on U.V-T. bacteria
(44)
(41)
(56)
(56)
=n
BP
TP
BP
TP
day 12
day 18
D
Fraction survival
1.00
0.75
0.50
0.25
0.00
0
10
20
30
40
Time (days)
Control
Carbenicillin
E
0.6903
0.0897
0.6152
WT on
Control
Carbenicillin
(71)
(76)
(89)
(84)
(47)
(60)
=n
4
4
15
15
18
24
Time (days)

Supplementary Figure 6. Nutritional and antibiotic regulation of host stiffness.

A) Mechanical properties as Young's Modulus (YM; kPa) of WT *C. elegans* grown on Bactopeptone/ agar lot 1 BP/A1 (blue), Bactopeptone /agar lot 2 BP/A2 (purple), Bactotryptone/ agar lot 1 TP/A1 (black) or soy protein/ agar lot 1 SP/A1 at chronological age of day 1 and day 12. Error bars indicate 95% CI, dotted line marks mean YM at day 1 for control condition (BP/A1). Two-tailed unpaired t-test for statistical comparison of control to conditions at day 1 (black), and control to conditions at day 12 (blue). **B)** Mechanical properties as YM (kPa) of WT *C. elegans* grown on control Bactopeptone medium (blue) or Bactotryptone medium (black) fed control (untreated) or heat-treated bacteria at chronological age day 12 and 18. Error bars indicate 95% CI. Two-way ANOVA Tukey's multiple comparison test for statistical comparison and interaction of terms (green) and to their respective untreated controls (black for day 12 and blue for day 18). **C**) Mechanical properties as YM (kPa) of WT *C. elegans* grown on U.V.-treated bacteria in control Bactopeptone medium (blue) or Bactotryptone medium (black) at chronological age day 12 and 18. Error bars indicate 95% CI. Two-tailed unpaired t-test for statistical comparison between control and Bactotryptone medium at same age. **D)** Lifespan curve of WT *C. elegans* grown on untreated control (blue) or carbenicillin-treated (red) plates (n = 90 and 102 respectively; log rank test $p < 0.001$ vs untreated control bacteria) and **E**) mechanical properties as YM (kPa) at chronological age day 4 and day 15, and at mean lifespan (D18 for control and D24 for carbenicillin). Two-tailed unpaired t-test for statistical comparison of control to treated conditions at same age.

n represented above the graph, show number of biologically independent worm samples; For lifespan measurements, *n* represents the number of worms scored as dead. For a summary of YM values and additional statistics for independent trials see Supplementary Data 1 and for a summary of worm lifespan trials and statistical comparison between genotypes see Supplementary Data 2. Source data are provided as a Source Data file.

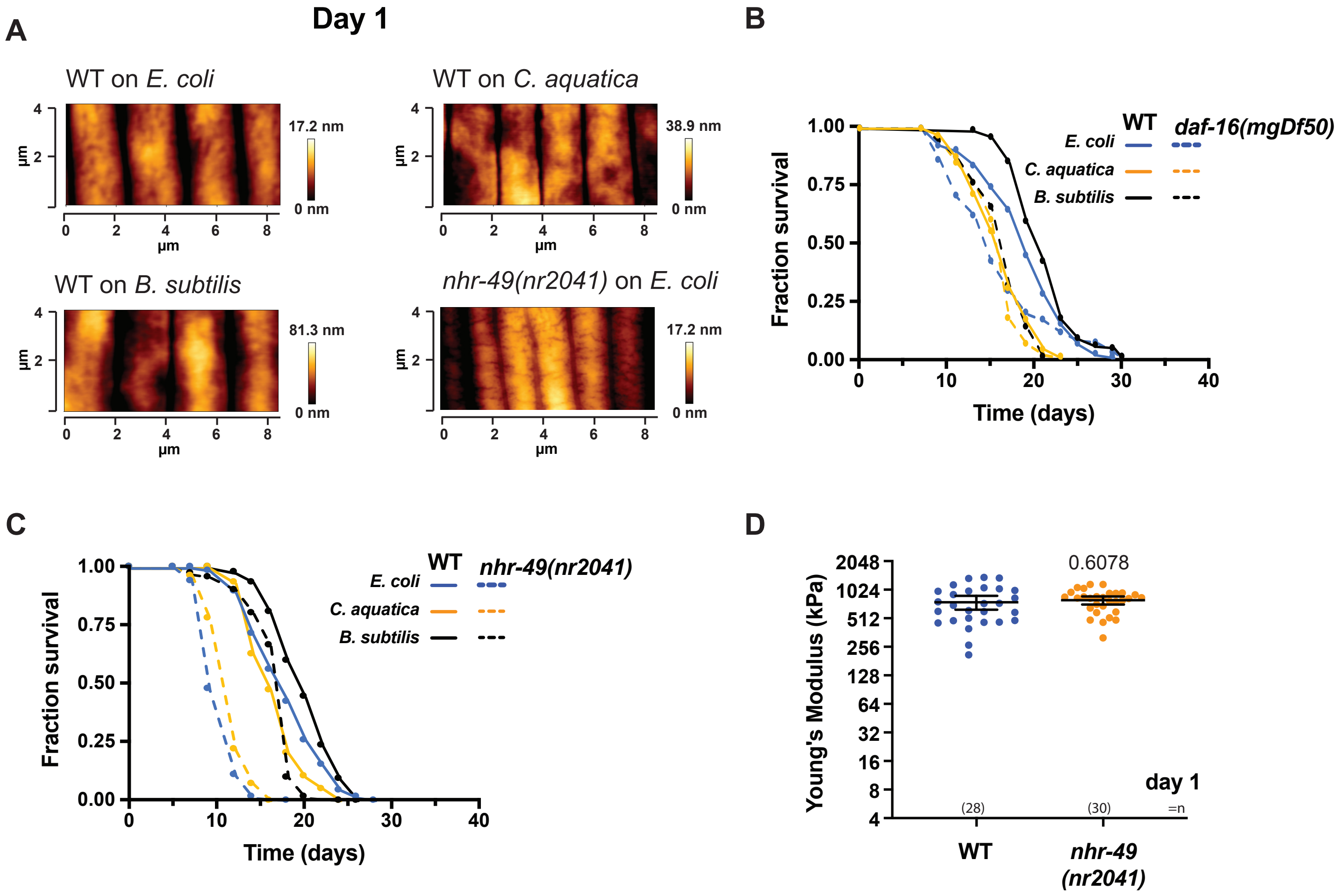

A
Day 1
WT on E. coli
17.2 nm
0 nm
WT on C. aquatica
38.9 nm
0 nm
WT on B. subtilis
81.3 nm
0 nm
nhr-49(nr2041) on E. coli
17.2 nm
0 nm
µm
B
Fraction survival
Time (days)
WT
daf-16(mgDf50)
E. coli
C. aquatica
B. subtilis
C
Fraction survival
Time (days)
WT
nhr-49(nr2041)
E. coli
C. aquatica
B. subtilis
D
Young's Modulus (kPa)
0.6078
day 1
(28)
(30)
=n
WT
nhr-49
(nr2041)

Supplementary Figure 7. Effects of bacterial diet and host genetics on young *C. elegans*.

A) Representative AFM cuticle topography images of 1-day-old wild type WT *C. elegans* grown on *E. coli*, *C. aquatica* or *B. subtilis*, and *nhr-49* mutant *C. elegans*. **B)** Lifespan curve of WT and *daf-16(mgDf50)* mutant *C. elegans* grown on OP50, *C. aquatica* and *B. subtilis* (see Supplementary Data 2 for statistical tests). **C)** Lifespan curve of WT and *nhr-49(nr2041)* mutant *C. elegans* grown on OP50, *C. aquatica* and *B. subtilis* (see Supplementary Data 2 for statistical tests). **D)** Mechanical properties as YM (kPa) of WT (blue) and *nhr-49* mutant (yellow) *C. elegans* at chronological age day1. Error bars indicate 95% CI. Two-tailed unpaired t-test for statistical comparison of WT to mutant.

n, represented above the graph show number of biologically independent worm samples. Source data are provided as a Source Data file.

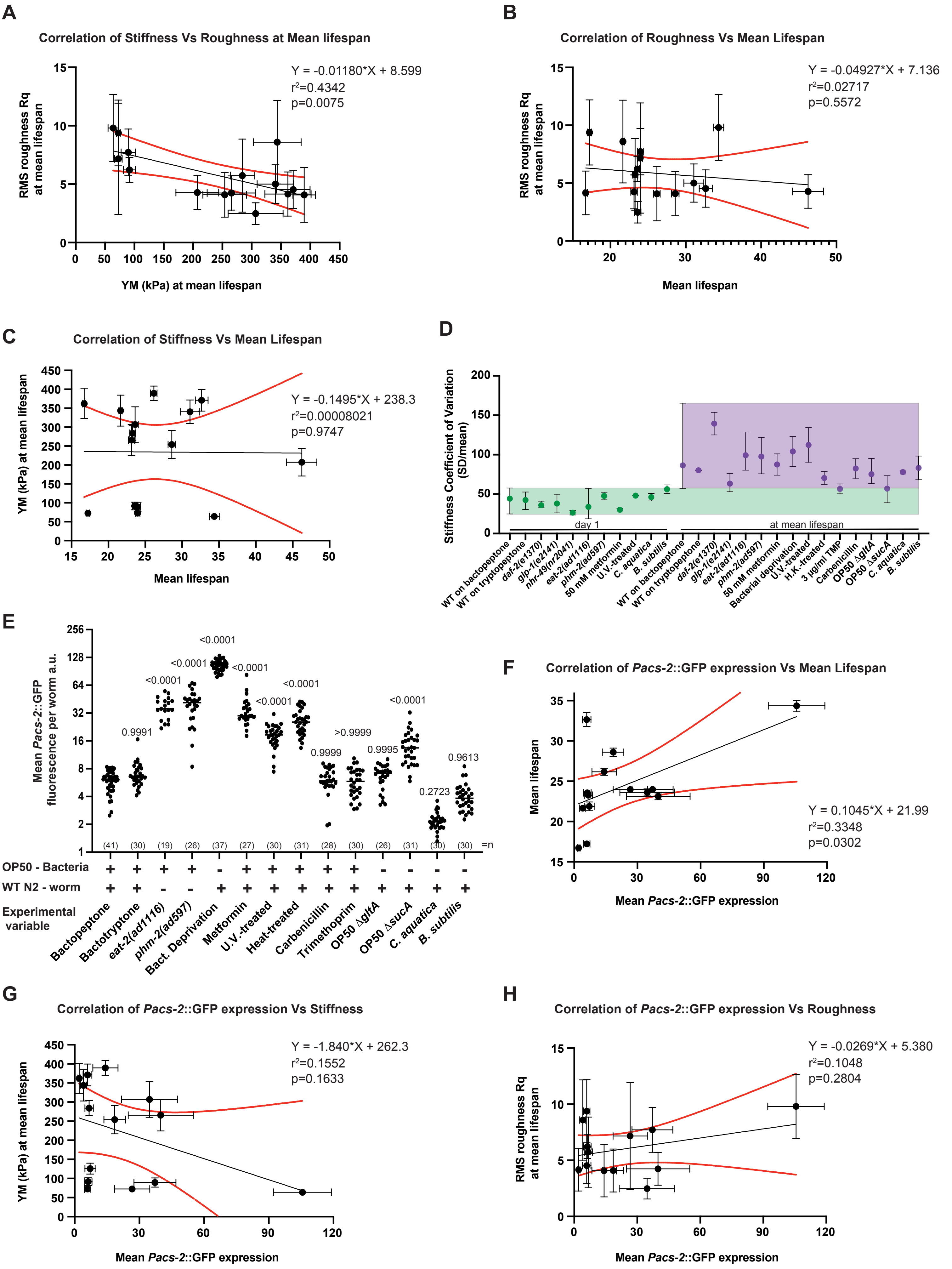
A
Correlation of Stiffness Vs Roughness at Mean lifespan
Y = -0.01180*X + 8.599
r²=0.4342
p=0.0075
RMS roughness Rq at mean lifespan
YM (kPa) at mean lifespan
B
Correlation of Roughness Vs Mean Lifespan
Y = -0.04927*X + 7.136
r²=0.02717
p=0.5572
RMS roughness Rq at mean lifespan
Mean lifespan
C
Correlation of Stiffness Vs Mean Lifespan
Y = -0.1495*X + 238.3
r²=0.00008021
p=0.9747
YM (kPa) at mean lifespan
Mean lifespan
D
Stiffness Coefficient of Variation (SD/mean)
day 1
at mean lifespan
WT on bactopeptone
WT on tryptopeptone
daf-2(e1370)
glp-1(e2141)
nhr-49(nr2041)
eat-2(ad1116)
phm-2(ad597)
50 mM metformin
U.V.-treated
C. aquatica
B. subtilis
WT on bactopeptone
WT on tryptopeptone
daf-2(e1370)
glp-1(e2141)
eat-2(ad1116)
phm-2(ad597)
50 mM metformin
Bacterial deprivation
U.V.-treated
H.K.-treated
3 ug/ml TMP
Carbenicillin
OP50 ΔgltA
OP50 ΔsucA
C. aquatica
B. subtilis
E
Mean Pacs-2::GFP fluorescence per worm a.u.
0.9991
<0.0001
<0.0001
<0.0001
<0.0001
<0.0001
<0.0001
0.9999
>0.9999
0.9995
<0.0001
0.2723
0.9613
(41) (30) (19) (26) (37) (27) (30) (31) (28) (30) (26) (31) (30) (30) =n
OP50 - Bacteria
WT N2 - worm
Experimental variable
Bactopeptone
Bactotryptone
eat-2(ad1116)
phm-2(ad597)
Bact. Deprivation
Metformin
U.V.-treated
Heat-treated
Carbenicillin
Trimethoprim
OP50 ΔgltA
OP50 ΔsucA
C. aquatica
B. subtilis
F
Correlation of Pacs-2::GFP expression Vs Mean Lifespan
Y = 0.1045*X + 21.99
r²=0.3348
p=0.0302
Mean lifespan
Mean Pacs-2::GFP expression
G
Correlation of Pacs-2::GFP expression Vs Stiffness
Y = -1.840*X + 262.3
r²=0.1552
p=0.1633
YM (kPa) at mean lifespan
Mean Pacs-2::GFP expression
H
Correlation of Pacs-2::GFP expression Vs Roughness
Y = -0.0269*X + 5.380
r²=0.1048
p=0.2804
RMS roughness Rq at mean lifespan
Mean Pacs-2::GFP expression

Supplementary Figure 8. Correlation between stiffness, cuticle decay, lifespan and *Pacs-2*::GFP expression.

A) Correlation between cuticle senescence measured as RMS roughness at mean lifespan and stiffness as Young's Modulus (YM; kPa) at mean lifespan. **B)** Correlation between cuticle senescence measured as RMS roughness at mean lifespan and mean lifespan. **C)** Correlation between stiffness as Young's Modulus (YM; kPa) at mean lifespan and mean lifespan. Conditions displayed in A, B and C are WT on OP50, without OP50 (BD), OP50 Δ*gltA*, OP50 Δ*sucA*, *B. subtilis*, *C. aquatica,* Heat- and U.V.-treated OP50, carbenicillin, trimethoprim and metformin treated OP50, and *phm-2*, *eat-2*, *glp-1*, *daf-2* worm mutants grown on OP50. All grown on NGM plates containing Bactopeptone. Linear regression was applied to the data and the best fit equation, goodness of fit (r^2) and p-value were calculated. Error bars represent S.D for roughness, and S.E.M. for stiffness and lifespan. **D)** Ageing increases heterogeneity in stiffness measurements. Coefficient of variation was calculated from stiffness measurements (YM) at day 1 and at mean lifespan for the conditions indicated in the Figure's x-axis. Error bars represent min and max values of independent experimental trials and shaded area represent min and max values of WT worms on Bactopeptrone on day 1 (green) or at mean lifespan day 18 (purple). **E)** Worm *Pacs-2*::GFP expression is increased in DR conditions (Bacterial deprivation and metformin) and DR mutants *eat-2* and *phm-2.* One-way ANOVA Dunnett's multiple comparison test was used for statistical comparison of WT worms on OP50 Bactopeptone to the other conditions indicated in the x-axis. *n* shows number of worms measured. **F)** Correlation between mean *Pacs-2*::GFP values and mean lifespan. **G)** Correlation between mean *Pacs-2*::GFP values and stiffness mechanical properties as Young's Modulus (YM; kPa) at mean lifespan. **H)** Correlation between mean *Pacs-2*::GFP values and cuticle senescence measured as RMS roughness at mean lifespan. Conditions displayed in F, G and H are displayed in the x-axis of E. Linear regression was applied to the data and the best fit equation, goodness of fit (r^2) and p-value were calculated. Error bars represent S.D. for *Pacs-2*::GFP and roughness, S.E.M for lifespan and stiffness. Source data are provided as a Source Data file.

Condition/treatment	Bacterial Physiology parameter (vs OP50 Bactopeptone (BP))			Fold change of mean YM at chronological lifespan (vs OP50 BP)	Fold change of mean YM at mean lifespan (vs OP50 BP)	References
	Proliferation (growth rate h-1 +/- SD)	Growth (AUC)	Metabolism (Dim 1 +/-SD; Dim 2 +/- SD)			
**OP50 Bactopeptone**	control (1.84 +/- 0.16)	control (0.40 +/- 0.040)	control (8.8+1.2 ; -6.8+1.0)	1	1	
**OP50 on Metformin**	impaired (1.32 +/- 0.14)	impaired (0.23 +/- 0.015)	altered (Pryor et al., 2019)	5.71	5.54	Cabreiro et al., 2013; Pryor et al., 2019; This study
**OP50 Trimethoprim**	impaired (1.47 +/-0.15)	impaired (0.31 +/- 0.014)	altered (-7.3+0.2 ; 0.4+0.3)	5.01	3.04	This study
**OP50 Carbenicillin**	no proliferation (0)	no growth (0)	not altered (8.4+0.9 ; -5.8+0.5)	1.36	1.09	This study
**OP50 U.V.-treated**	no proliferation (0)	no growth (0)	altered (-8.1+0.8 ; 0.0+0.3)	4.20	3.67	This study
**OP50 Heat-treated**	no proliferation (0)	no growth (0)	strongly altered (-14.0+0.2 ; 1.1+0.2)	0.09	1.05	This study
**OP50 Tryptopeptone**	not impaired (1.69 +/- 0.38)	not impaired (0.67 +/- 0.017)	slightly altered (9.6+0.9 ; 14.3+0.4)	5.00	1.60	This study
**OP50 Δ*gltA***	not impaired (1.74 +/- 0.13)	impaired (0.29 +/- 0.007)	slightly altered (2.7+0.8 ; -3.2+0.3)	3.77	2.78	This study
**OP50 Δ*sucA***	not impaired (1.74 +/- 0.14)	strongly impaired (0.19 +/- 0.037)	not tested	8.31	3.81	This study

Supplementary Table 1. Qualitative and quantitative analysis of treatments affecting bacterial physiology parameters and worm stiffness. Bacterial proliferation parameters measured as growth rate h^{-1} as a function of population OD doubling time during exponential phase; total growth measured as area under the curve as a function of OD over time; and metabolomic alterations in bacteria indicated by the values in the first two components in the PCA plots. Fold change in worm stiffness (Young's Modulus) upon specified treatment compared to worms in the control condition (OP50) at both chronological age and mean lifespan.
