# Supplementary material for: Mechanical properties measured by atomic force microscopy define health biomarkers in ageing C. elegans
Source: Nat Commun. 2020 Feb 25;11:1043. doi: 10.1038/s41467-020-14785-0 (PMC7042263; doi:10.1038/s41467-020-14785-0)
Supplement: Supplementary file 3 — Description of Additional Supplementary Information [file 41467_2020_14785_MOESM3_ESM.pdf]

Description of Additional Supplementary Files

File Name: Supplementary Data 1
Description: Contains data and statistical tests associated with AFM experiments.

File Name: Supplementary Data 2
Description: Contains data and statistical tests associated with C. elegans lifespan experiments.

File Name: Supplementary Data 3
Description: Contains data associated with E. coli metabolomics experiments from Supplementary Figure 5.
