# Supplementary material for: Mechanical properties measured by atomic force microscopy define health biomarkers in ageing C. elegans
Source: Nat Commun. 2020 Feb 25;11:1043. doi: 10.1038/s41467-020-14785-0 (PMC7042263; doi:10.1038/s41467-020-14785-0)
Supplement: Supplementary file 7 — Reporting Summary [file 41467_2020_14785_MOESM7_ESM.pdf]

nature research

Corresponding author(s): Filipe Cabreiro and Clara Essmann

Last updated by author(s): Sep 25, 2019

Reporting Summary

Nature Research wishes to improve the reproducibility of the work that we publish. This form provides structure for consistency and transparency in reporting. For further information on Nature Research policies, see Authors & Referees and the Editorial Policy Checklist .

Statistics

For all statistical analyses, confirm that the following items are present in the figure legend, table legend, main text, or Methods section.

n/a	Confirmed	
☐	☒	The exact sample size (*n*) for each experimental group/condition, given as a discrete number and unit of measurement
☐	☒	A statement on whether measurements were taken from distinct samples or whether the same sample was measured repeatedly
☐	☒	The statistical test(s) used AND whether they are one- or two-sided<br>*Only common tests should be described solely by name; describe more complex techniques in the Methods section.*
☒	☐	A description of all covariates tested
☐	☒	A description of any assumptions or corrections, such as tests of normality and adjustment for multiple comparisons
☐	☒	A full description of the statistical parameters including central tendency (e.g. means) or other basic estimates (e.g. regression coefficient) AND variation (e.g. standard deviation) or associated estimates of uncertainty (e.g. confidence intervals)
☐	☒	For null hypothesis testing, the test statistic (e.g. *F*, *t*, *r*) with confidence intervals, effect sizes, degrees of freedom and *P* value noted<br>*Give P values as exact values whenever suitable.*
☒	☐	For Bayesian analysis, information on the choice of priors and Markov chain Monte Carlo settings
☒	☐	For hierarchical and complex designs, identification of the appropriate level for tests and full reporting of outcomes
☐	☒	Estimates of effect sizes (e.g. Cohen's *d*, Pearson's *r*), indicating how they were calculated

Our web collection on statistics for biologists contains articles on many of the points above.

Software and code

Policy information about availability of computer code

Data collection	We used the AFM NanoWizard3 (JPK) to acquire force-indentation and topography data. Worm imaging was performed using a Zeiss Axioplan V16 dissecting microscope and captured using the ZEN pro software (Carl Zeiss) or using a Leica DMRXA2 microscope and Orca digital camera (Hamamatsu) and captured using Volocity 6.3 Software. Bacterial growth data was acquired using a Tecan Infinite M200 PRO microplate reader and Magellan V6.5 software.
Data analysis	AFM data were analysed using JPK analysis software version 5. Folate mass spectrometric data were analysed using MassLynx Software (Waters). For untargeted metabolomics data peaks detected in CE-TOFMS analysis were extracted using MasterHands ver.2.17.1.11 automatic integration software (Keio University) in order to obtain peak information including m/z, migration time (MT), and peak area. Further analysis of the metabolomics data was performed by R programming language (v. 3.5.1), where the tidyverse package (v. 1.2.1) was used to work with the datasets, FactoMineR package (v. 1.41) was used to analyse PCA, and ComplexHeatmap package (v. 1.20) was used to represent the complete heatmap. Bacterial growth data was analyzed using R (R Core Team) and Area Under the Curve was calculated with the function auc from the pROC library in R (v. 1.14.0). Worm imaging data was processed using ImageJ. Remaining data analysis was performed using GraphPad Prism software (V8). Post-hoc analysis was performed using the online calculator htpps://clincalc.com/stats/power.aspx.

For manuscripts utilizing custom algorithms or software that are central to the research but not yet described in published literature, software must be made available to editors/reviewers. We strongly encourage code deposition in a community repository (e.g. GitHub). See the Nature Research guidelines for submitting code & software for further information.

Data

Policy information about availability of data

All manuscripts must include a data availability statement. This statement should provide the following information, where applicable:

- Accession codes, unique identifiers, or web links for publicly available datasets
- A list of figures that have associated raw data
- A description of any restrictions on data availability

We have provided in Supplementary Table S1 all analysed AFM data, in Table S2 all analysed lifespan data and in Table S3 all analysed metabolomics data. All source data for these datasets are available in the source data file. Further information and reasonable requests for resources and reagents should be directed to and will be fulfilled by the corresponding authors.

Field-specific reporting

Please select the one below that is the best fit for your research. If you are not sure, read the appropriate sections before making your selection.

☒ Life sciences ☐ Behavioural & social sciences ☐ Ecological, evolutionary & environmental sciences

For a reference copy of the document with all sections, see nature.com/documents/nr-reporting-summary-flat.pdf

Life sciences study design

All studies must disclose on these points even when the disclosure is negative.

Sample size	Lifespan, metabolomics and bacterial growth sample size data in this study was determined based on the significance obtained from previous studies with similar experimental setups. AFM data was determined practically, based on the experimental design (e.g age) and resource constraints.
Data exclusions	No data were excluded from this study.
Replication	All experiments were completed with multiple, consistent, biological replicates with at least n=3 or >3 for all conditions tested. AFM experiments were replicated independently 2-4 times depending on the effect size of the intervention versus control condition.
Randomization	Not relevant to this study.
Blinding	Authors were partially blind to the experimental conditions between the ageing of the worm cohorts and the AFM measurements (performed by separate experimentalists). Similarly, for the metabolomics data, the extractions and measurements of metabolites in the samples were performed without prior knowledge of the biological meaning of the data. Computational analysis of the data was also performed without prior knowledge of the biological meaning of the data.

Reporting for specific materials, systems and methods

We require information from authors about some types of materials, experimental systems and methods used in many studies. Here, indicate whether each material, system or method listed is relevant to your study. If you are not sure if a list item applies to your research, read the appropriate section before selecting a response.

Materials & experimental systems

n/a	Involved in the study	
☒	☐	Antibodies
☒	☐	Eukaryotic cell lines
☒	☐	Palaeontology
☐	☒	Animals and other organisms
☒	☐	Human research participants
☒	☐	Clinical data

Methods

n/a	Involved in the study	
☒	☐	ChIP-seq
☒	☐	Flow cytometry
☒	☐	MRI-based neuroimaging

Animals and other organisms

Policy information about studies involving animals; ARRIVE guidelines recommended for reporting animal research

Laboratory animals	Caenorhabditis elegans of various genotypes and ages were analysed in this manuscript. All data was collected with hermaphrodites.
Wild animals	Not applicable.

Field-collected samples	Not applicable.
Ethics oversight	Not applicable.

Note that full information on the approval of the study protocol must also be provided in the manuscript.
